# Impact of Temperature on Phenolic and Osmolyte Contents in In Vitro Cultures and Micropropagated Plants of Two Mediterranean Plant Species, *Lavandula viridis* and *Thymus lotocephalus*

**DOI:** 10.3390/plants11243516

**Published:** 2022-12-14

**Authors:** Inês Mansinhos, Sandra Gonçalves, Raquel Rodríguez-Solana, José Luis Ordóñez-Díaz, José Manuel Moreno-Rojas, Anabela Romano

**Affiliations:** 1 MED—Mediterranean Institute for Agriculture, Environment and Development & CHANGE—Global Change and Sustainability Institute, Faculdade de Ciências e Tecnologia, Universidade do Algarve, Campus de Gambelas, 8005-139 Faro, Portugal; 2 Department of Agroindustry and Food Quality, Andalusian Institute of Agricultural and Fisheries Research and Training (IFAPA), Avenida Menendez-Pidal, SN, 14004 Córdoba, Spain; 3 Foods for Health Group, Instituto Maimónides de Investigación Biomédica de Córdoba (IMIBIC), Avenida Menendez-Pidal, SN, 14004 Córdoba, Spain

**Keywords:** abiotic stress, osmolytes, shikimate/phenylpropanoid pathway, green extract, NADES, antioxidant activity, phenolic acids

## Abstract

In this study, in vitro cultures and micropropagated plants of two Mediterranean aromatic plants, *Lavandula viridis* L’Hér and *Thymus lotocephalus* López and Morales, were exposed to different temperatures (15, 20, 25, and 30 °C). The effect of temperature on the levels of hydrogen peroxide (H_2_O_2_), lipid peroxidation, and osmoprotectants (proline, soluble sugars, and soluble proteins), as well as on the phenolic profile by HPLC-HRMS and intermediates of the secondary metabolism (phenylalanine ammonia lyase (PAL) activity and shikimic acid content), was investigated. Moreover, the antioxidant activity of the plant extracts was also analyzed. Overall, considering the lipid peroxidation and H_2_O_2_ content, the extreme temperatures (15 and 30 °C) caused the greatest damage to both species, but the osmoprotectant response was different depending on the species and plant material. In both species, phenolic compounds and related antioxidant activity increased with the rise in temperature in the micropropagated plants, while the opposite occurred in in vitro cultures. *L. viridis* cultures showed the highest biosynthesis of rosmarinic acid (92.6 g/kg_DW_) at 15 °C and seem to be a good alternative to produce this valuable compound. We conclude that contrasting temperatures greatly influence both species’ primary and secondary metabolism, but the response is different depending on the plant micropropagation stage.

## 1. Introduction

Lamiaceae is a large family of flowering plants widely distributed in different regions (mostly in the Mediterranean and Asia) and with great economic value. Traditionally, these plants are used as culinary herbs, shrubs, ornamentals, and medicinal plants. *Thymus* and *Lavandula* are two important aromatic genera within this family that produce important phytochemicals, namely phenolic and volatile compounds. *Thymus lotocephalus* López and Morales and *Lavandula viridis* L’Hér are two Mediterranean species endemic to the south of Portugal (Algarve) and the Iberian Peninsula, respectively, which present promising biological properties, such as pathological-enzyme inhibiting capacity, antioxidant activity, and ultraviolet protection [1,2,3,4]. Recent studies showed that in vitro-produced cultures of these species can be a good alternative to produce phenolic compounds [4,5,6]. In vitro culture which is performed under controlled conditions allows the large-scale production of homogenous plant material independently of geographical and seasonal variations. The process of producing micropropagated plants through in vitro culture systems involves different sequential phases occurring in the first stages in vitro (initiation, multiplication, and rooting) and at the end of the process already in ex vitro conditions (acclimatization). This technique has been considered adequate to produce secondary metabolites without compromising the species’ natural populations [5,6,7], and it is even possible to enhance the production of these compounds by manipulating the culture conditions [8], such as medium composition or environment (e.g., light, temperature, quality, etc.) [9]. In vitro culture is also described as a good tool to investigate plant response to abiotic stress factors [8]. Given that each plant species presents its optimal temperature limits, when growing out of these ranges physiological and biochemical functions can be affected.

Adverse environmental factors such as extreme temperatures (cold and heat), drought, ultraviolet radiation, and soil contamination are the main abiotic factors that negatively affect physiology, as well as the primary and secondary metabolism of plants [10,11]. While primary metabolism, constituted by biomolecules (sugars, proteins, vitamins, nucleic acid components, and lipids), plays a direct role in plant metabolism, development, and growth [12], secondary metabolism includes metabolites that are non-essential but confer stress protection, increasing plants’ fitness [13]. The up-or down-regulation of certain primary and secondary metabolites is one of the main mechanisms adopted by plants to tolerate abiotic stresses [10]. Soluble sugars, soluble proteins, and proline present an essential function as osmolytes and their accumulation increases plant survival under stress by maintaining the water status of the cells, biomolecules and lipid membranes subjected to dehydration, suppressing photorespiration, acting as antifreeze, inhibiting the reactive oxygen species (ROS) production and maintaining redox balance, preserving intracellular potassium homeostasis, and activating transcription factors responsible for stress responses [14,15,16]. Thus, the accumulation of these osmotic regulators, also called osmoprotectants, is considered a central mechanism in plant stress response [17].

Phenolic compounds, which are the largest group of secondary metabolites ranging from simple aromatic rings to more complex ones (e.g., lignins), are also involved in stress tolerance. These compounds exhibit a crucial role in several physiological mechanisms to increase a plant’s adaptability and exhibit a strong antioxidant capacity essential to scavenging ROS such as H_2_O_2_, OH˙^−^, O_2_˙^−^, and ^1^O_2_ [18]. The precursors of most plant phenolics are synthesized mainly by the shikimic acid pathway, with shikimic acid biosynthesis the first step of this pathway. This is then converted into the next key intermediate, chorismite, which is responsible for the production of phenylalanine and tyrosine (the main precursors of the biosynthesis of phenolics through the phenylpropanoid pathway). In most plants, phenylalanine is the main substrate for the synthesis of phenolic compounds [19]. The first step of the phenylpropanoid pathway involves the conversion of phenylalanine to cinnamic acid through the phenylalanine-ammonia-lyase (PAL) enzyme, an important enzyme in phenolics biosynthesis.

Temperature is a primary factor that affects the rate of plant growth and development. Usually, plants grow better and produce higher biomass yields in less stressful conditions. On the other hand, when exposed to environmental stresses, such as extreme temperatures, plants generally enhance the secondary metabolites production to protect themselves from stress conditions [20]. This study aimed to compare the response of in vitro cultures (multiplication stage) and micropropagated plants (after acclimatization stage) of *L. viridis* and *T. lotocephalus* to different temperatures (15, 20, 25, and 30 °C) at the physiological and biochemical levels, as well as at the primary and secondary metabolism, with emphasis on phenolics biosynthesis. This is the first report investigating the temperature effect on *L. viridis* and *T. lotocephalus* species and the tested temperatures were selected based on previous studies on Lamiaceae species described in the literature [11,20,21]. Once plants respond differently to environmental stress, this study will be important to identify the specific mechanisms used by these two aromatic species to survive under a changing environment scenario, including the biosynthesis of the health-promoting bioactive compounds.

## 2. Results and Discussion

### 2.1. Evaluation of Photosynthetic Pigments and Oxidative Stress Markers

Temperature is one of the main factors that directly affects several physiological and biochemical processes in plants, such as photosynthesis, in which chlorophylls are critical for collecting light during this process. The photosynthetic pigments’ (total chlorophylls and carotenoids) accumulation in *T. lotocephalus* and *L. viridis* was stimulated by the lowest temperatures tested (Table 1).

In both species, photosynthetic pigment contents were higher when in vitro cultures and ex vitro micropropagated plants were exposed to 15 and 20 °C, respectively. Likewise, other authors presented similar results, as is the case of *Thymus transcaucasicus* Ronn., in which plants were exposed to 15, 20, and 25 °C and achieved the highest carotenoids and total chlorophyll accumulation under 20 °C [11]. On the contrary, *Rosmarinus officinalis* L. (Lamiaceae) [22], *Brassica rapa* L. (Brassicaceae) [23], and *Solanum lycopersicum* L. (Solanaceae) [14] plants showed significant decreases in chlorophyll and carotenoid contents when exposed to the coldest temperatures (4–12 °C). In this study, the highest temperature (30 °C) caused a significant decline in total chlorophylls (65–81%) and carotenoid contents (65–68%) in in vitro cultures and ex vitro micropropagated plants of both species, compared to the contents achieved at 15 and 20 °C. The same was found in other plants under heat stress [24,25,26]. Heat stress disrupts the structure and function of thylakoid membranes within the chloroplast, decreasing chlorophyll content and photosynthesis. The membrane integrity loss may be mainly due to ROS production resulting from photorespiration, which leads to protein degradation and reduces CO_2_ assimilation and photosynthetic efficacy [24,26]. The loss of membrane integrity under heat stress was confirmed by the highest levels of lipid peroxidation, estimated by MDA content, in both cultures and plants of *L. viridis* and *T. lotocephalus* exposed to 30 °C. Higher MDA levels in plants exposed to heat stress (>35 °C) were also reported by several authors [24,26,27]. In this study, overall, there were no significant differences between the MDA contents obtained at 20 and 15 °C. However, an exception was observed in *T. lotocephalus* ex vitro plants in which lipid peroxidation was higher at 15 °C. Similarly, Luis et al. [22] reported that in *R. officinalis*, the temperature of 12 °C caused an increase in MDA content compared to 22 °C. On the other hand, the results of Kalisz et al. [28] demonstrated that MDA concentration was higher in leaves of *Salvia officinalis* L. treated at 18 °C than at 4 °C. In this study, the temperature of 20 °C caused the smallest production of H_2_O_2_ and the content of this ROS increased under extreme temperatures (15 and 30 °C), especially 15 °C (9.63 ± 0.18 µmol/g_FW_ for *L. viridis* and 7.99 ± 0.66 µmol/g_FW_ for *T. lotocephalus*). These results were consistent with those reported by other authors in plants subjected to cold [14,22,23] or heat stresses [26,27,29]. When *Melissa officinalis* L., another aromatic and medicinal plant belonging to the Lamiaceae family, was subjected to short-term heat stress (38 °C for 5 h), the H_2_O_2_ content increased exponentially during the first two hours. However, after this period plants have probably activated different oxidative stress processing systems in response to ROS and, consequently, decreased their production [27]. In this study, comparing cultures and plants independently of temperature treatment, plants showed higher H_2_O_2_ production (190–390%) in both species. These results were expected given the different growth conditions (culture media *vs* substrate) between in vitro cultures and micropropagated plants growing in ex vitro conditions. Moreover, during in vitro conditions, plantlets grow under special conditions, and after ex vitro transfer, these plantlets are subjected to higher irradiance and lower air humidity [30].

### 2.2. Primary Metabolites as Osmoprotectants

Soluble sugars, essential as structural and metabolic supplies for plant cells, also present an important role as osmoprotectants [10]. As shown in Table 1, the impact of temperature on total soluble sugar content was variable depending on the species and plant material but, in general, it was lower at the extreme temperature of 30 °C, especially in *T. lotocephalus* cultures which achieved the lowest value (28.9 ± 0.12 mg/g_FW_). These results were consistent with those obtained for other plants, in which heat stress also caused lower soluble sugar accumulation [21,25,31]. At 30 °C, cultures and plants triggered the highest lipid peroxidation, indicating stressful conditions, and the total soluble sugar content was generally low. This can be explained by the fact that in stressful conditions the content of sugars must be accumulated in higher proportion in roots instead of leaves [25]. In cultures of both species, sugar content was promoted by the lowest temperature tested (15 °C). Likewise, this temperature also enhanced the accumulation of sugars in other plants. Guo et al. [13] exposed *Ginkgo biloba* L. leaves to different temperature regimes (35/30, 25/20, and 15/10 °C, day/night) and the coldest one improved the accumulation of soluble sugars. Similarly, the sugar content was higher in in vitro cultures of *Alcantarea imperialis* (Carrière) Harms [32] and *Ajuga bracteosa* Wall. ex. Benth. [21] grown at 15 °C than at 30 °C. Likewise, other reports suggest chillier temperatures (5–10 °C) as a strategy to improve the accumulation of sugars [14,33].

Soluble proteins, besides providing a storage form of nitrogen, are considered another osmotic regulator of plants protecting them against abiotic stresses, especially heat and drought [24,34]. Overall, total soluble protein content was lower at 15 and 30 °C (Table 1). The exception was observed in *T. lotocephalus* cultures in which the highest accumulation of these osmolytes occurred at 30 °C (10.1 ± 0.81 mg/g_FW_). Similar results were obtained by other authors after subjecting plants to heat [24,35] or cold [23] stress conditions. 

In addition to the essential role of proline in primary metabolism as a component of proteins, this amino acid is one of the most commonly compatible solutes accumulated by plants under abiotic stresses [36]. In this study, both species accumulated the highest content of proline when cultures and plants were exposed to heat stress (30 °C). Additionally, cold also induced a positive effect on the levels of this osmoprotectant in *T. lotocephalus* cultures, achieving the highest accumulation at 15 °C (6.05 ± 0.31 µmol/g_FW_). In other species, high [15,26,37] and low temperatures [23] also enhanced the accumulation of proline. Interestingly, independently of the temperature, ex vitro plants of both species demonstrated much less free proline levels than in vitro cultures, given this accumulation is practically absent in the case of *T. lotocephalus*. Comparable results were reported by other authors who compared proline concentration in in vitro and ex vitro plants of walnut and obtained proline levels two times higher in in vitro than in ex vitro plants [38]. 

### 2.3. Secondary Metabolism Keys

#### 2.3.1. Shikimic Acid and PAL Activity

**Figure 1:**
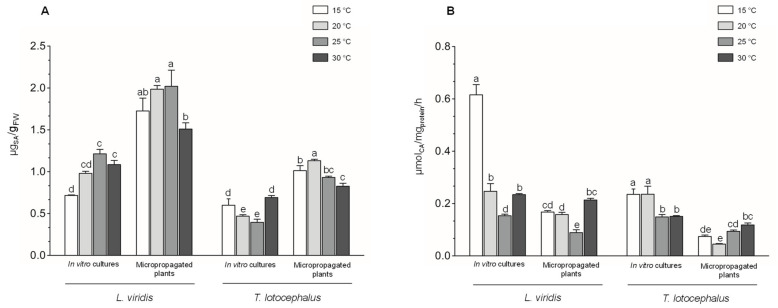
Effect of exposure to different temperatures (15, 20, 25, and 30 °C) on secondary metabolism keys—(**A**) shikimic acid content and (**B**) phenylalanine ammonia lyase (PAL) activity—of in vitro cultures and micropropagated plants of *Lavandula viridis* and *Thymus lotocephalus*. For each species, the results were analyzed by one-way analysis of variance (ANOVA) and the graph bars followed by different letters (a–d) are significantly different at *p* < 0.05 (Duncan’s New Multiple Range Test).

The results of shikimic acid content indicate that temperature changes secondary metabolic pathways in the studied species (Figure 1A). 

In general, the content of shikimic acid was lower at extreme temperatures (15 or 30 °C) in both species and plant material. This suggests the highest metabolic activity of the plants and cultures under extreme temperatures, indicating the use of this precursor for the biosynthesis of phenolics. The accumulation of shikimic acid was also reduced under other stress conditions in other species [39], particularly under high temperatures [40,41,42]. In both Lamiaceae species, the extreme temperatures positively upregulated the activity of PAL, suggesting the possible involvement of the phenylpropanoid pathway in stress tolerance (Figure 1B). PAL activity particularly increased in plants at 30 °C (0.12 ± 0.01 μmol/mg/h in *T. lotocephalus* and 0.21 ± 0.01 μmol/mg/h in *L. viridis*), contrary to cultures, in which the activity of this enzyme was significantly higher at the lowest temperature (0.24 ± 0.02 μmol/mg/h in *T. lotocephalus* and 0.62 ± 0.04 μmol/mg/h in *L. viridis*), especially in *L. viridis*. PAL activity was also improved at 15/10 °C and reduced at 35/30 °C in leaves of *G. biloba* [13]. Similarly, lower temperatures increased PAL activity in tomato [14,43] and oak plants [33]. This enzyme is the first one involved in the phenylpropanoid pathway and has an exceptionally critical role in starting the resistance mechanisms against stresses [39].

#### 2.3.2. Phenolic Contents, Phenolic Profile and Antioxidant Activity of the Extracts

**Figure 2:**
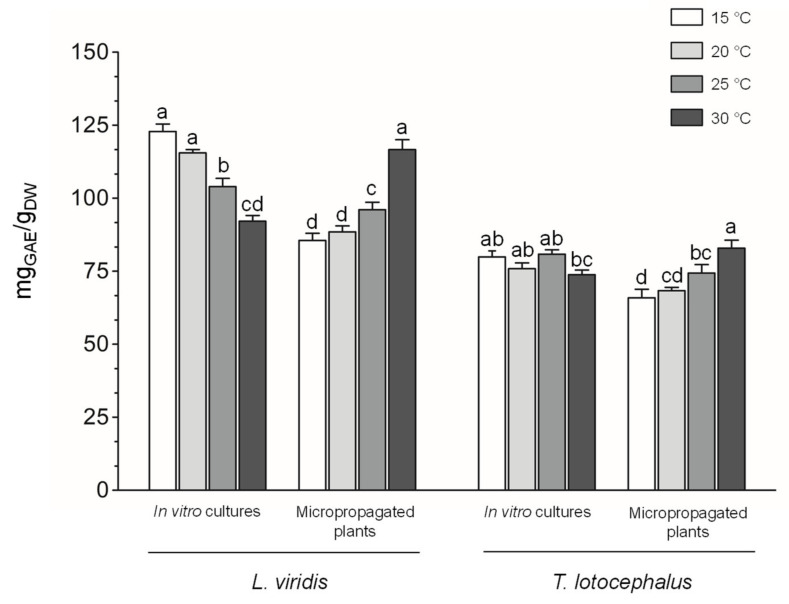
Effect of exposure to different temperatures (15, 20, 25, and 30 °C) on total phenolic content determined by F-C method of in vitro cultures and micropropagated plants of *Lavandula viridis* and *Thymus lotocephalus*. For each species, the results were analyzed by one-way analysis of variance (ANOVA) and the graph bars followed by different letters (a–d) are significantly different at *p* < 0.05 (Duncan’s New Multiple Range Test).

**Figure 3:**
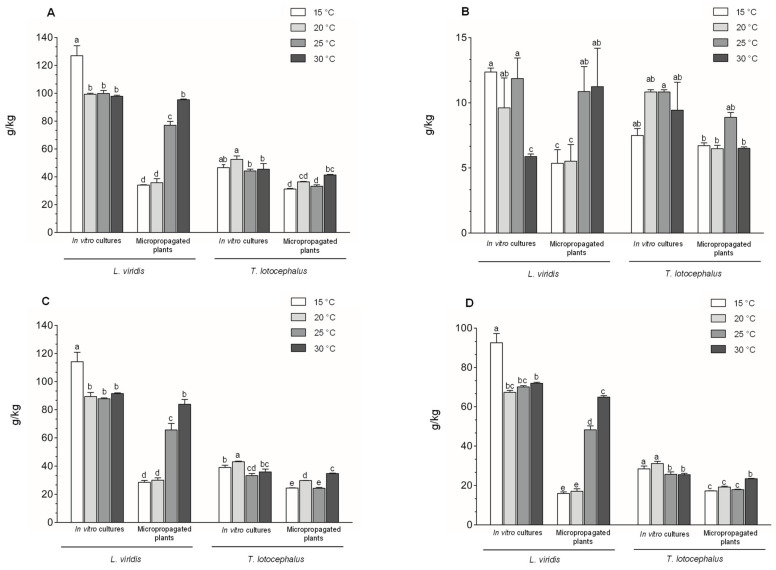
Effect of exposure to different temperatures (15, 20, 25, and 30 °C) on (**A**) total phenolic content, (**B**) total flavonoid content, (**C**) total phenolic acids content, and (**D**) total rosmarinic acid content determined by HPLC-HRMS of in vitro cultures and micropropagated plants of *Lavandula viridis* and *Thymus lotocephalus*. For each species, the results were analyzed by one-way analysis of variance (ANOVA) and the graph bars followed by different letters (a–e) are significantly different at *p* < 0.05 (Duncan’s New Multiple Range Test).

In the present study, total phenolic content was determined by F-C assay (Figure 2) and HPLC-HRMS (Figure 3A). 

The total phenolic content determined by HPLC-HRMS in plants of both species was greater at high temperatures, especially in *L. viridis* which produced almost three times more phenolics at 30 °C (95.3 ± 0.53 g/kg_DW_) compared to 15 °C (34.0 ± 0.31 g/kg_DW_) (Figure 3A and Table S3). Similarly, other authors have reported an increase in phenolic compounds accumulation at elevated temperatures [20,44]. Contrariwise, in *L. viridis* cultures, the highest biosynthesis of phenolics was observed at 15 °C. Similarly, cultures of *A. bracteosa*, another plant belonging to the Lamiaceae family, were exposed to 10, 15, 20, 25, and 30 °C and the maximum phenolics was also achieved at 15 °C [21]. According to the phenolic results, cold temperature seems to be more stressful to in vitro cultures, while heat temperature appears to cause more stress in plants. The different response and sensitivity to temperature between in vitro cultures and micropropagated plants, in terms of phenolics production, is probably related to their distinct growth specificities, and morphological and physiological characteristics. Cultures (shoots with aerial part only) are grown in vitro under totally controlled conditions in an artificial culture medium supplemented with a carbon source, and thus in heterotrophic conditions, in a closed culture vessel, at high air humidity and limited gas exchange conditions [30]. On the other hand, micropropagated plants (with root and aerial part) growing on pots with substrate are already fully acclimatized to ex vitro conditions and better mimic plants under in vivo conditions.

Regarding the phenolic profile of the extracts, a total of thirty-one phenolic compounds (twenty-four phenolic acids, five flavonoids, a coumarin derivative, and a hydroxybenzaldehyde) were identified in both Lamiaceae species; however, only twenty-four and twenty-three were detected in quantifiable amounts in *T. lotocephalus* and *L. viridis* extracts, respectively. Total flavonoids and phenolic acids, quantified by HPLC-HRMS, are presented in Figure 3B,C, respectively, and described in detail in Supplementary Tables S3 and S4. *T. lotocephalus* cultures (10.8 ± 0.17 g/kg_DW_) and plants (8.89 ± 0.37 g/kg_DW_) showed the highest contents of flavonoids at 25 °C. Regarding the total phenolic acids, in this species, the maximum amounts were achieved at 20 °C in the cultures (43.0 ± 0.42 g/kg_DW_) and at 30 °C in the plants (34.7 ± 0.32 g/kg_DW_). In *L. viridis*, the highest production of flavonoids and phenolic acids was achieved at the lowest temperature (15 °C) in the cultures and at the highest (30 °C) in the plants. Also, Guo et al. [13] reported that in leaves of *G. biloba* flavonoid contents increased or decreased under 15/10 °C or 35/30 °C, respectively. In agreement with previous studies on these species [5,6], rosmarinic acid (RA) was the major phenolic compound identified in both species (Figure 3D), representing 68–74%, 47–68%, 56–61%, and 52–57% of the total phenolics in *L. viridis* cultures, *L. viridis* plants, *T. lotocephalus* cultures, and *T. lotocephalus* plants, respectively (Supplementary Tables S3 and S4). *L. viridis* revealed a much higher content of RA than *T. lotocephalus*, especially under in vitro conditions, in which it biosynthesized the double content in all tested temperatures. Indeed, *L. viridis* cultures were shown to be a better approach to produce RA (67.3–92.6 g/kg_DW_) than micropropagated plants growing in ex vitro conditions (15.9–64.9 g/kg_DW_). The highest biosynthesis of this phenolic acid (92.6 g/kg_DW_) occurred when cultures were exposed to 15 °C. Comparatively to other studies, this is the greatest RA content achieved in cultures from this species [5,6,45]. Additionally, the maximum level of RA reported in *L. viridis* wild plants was 38.8 g/kg_extract_ [2,45], which is 2.4 times lower than that obtained in this study in in vitro cultures. These results are very remarkable given the advantages offered by in vitro culture for plant biomass and secondary metabolites production [7]. There are some studies in the literature testing the effect of temperature on the production of RA and the results are variable. The impact of different constant temperatures (15, 20, 25 °C) on the production of bioactive compounds from *T. transcaucasicus* plants was studied and the highest RA content (~7.5 g/kg_DW_) was achieved at 25 °C, which was 2.3–4.1 times lower than the RA contents obtained in our study for *T. lotocephalus* [11]. *Melissa officinalis* L. hydroponic cultures were subjected to short-term heat stress (38 °C, 5 h) and a significant increase in RA content (13 mg/g_FW_) was observed [27]. On the other hand, seven chemotypes of *Mentha spicata* L. were exposed to constant heat (30 °C) for 4 weeks and a complete loss of RA in all samples was observed [46]. In another study, two accessions of *R. officinalis* plants were exposed to cold stress (12/6 °C), showing different responses with a 50% increase in RA production for one accession and a 50% RA decrease for the other [22]. These variable results suggest that RA production depends on temperature, and is also affected by other parameters, such as plant species, genotype, plant material and propagation methods.

In the present study, the second most abundant compounds were epigallocatechin gallate and methyl 6-O-galloyl-*β*-D-glucopyranoside, in both species, although without significant differences between plant material types (Supplementary Tables S3 and S4). As far as we know, this is the first study identifying the phenolic acids salvianolic acid F, methyl 6-O-galloyl-*β*-D-glucopyranoside, and melitric acid B, and the flavonoids epigallocatechin gallate, theaflavic acid, and dihydromorelloflavone in *Lavandula* genera. The phenolic acids sagerinic acid, salviaflaside, and methylrosmarinic acid, as well as the hydroxybenzaldehyde protocatechuic aldehyde, even though being identified for the first time in *L. viridis*, were found in other *Lavandula* species (*L. pedunculata*, *L. dentata*, and *L. stoechas)* [47,48]. The identification of these new compounds may be related to the stressful growth conditions and/or extraction conditions, namely the use of a green solvent. In *T. lotocephalus*, all these these biocompounds were previously identified by our group [4]. 

**Figure 4:**
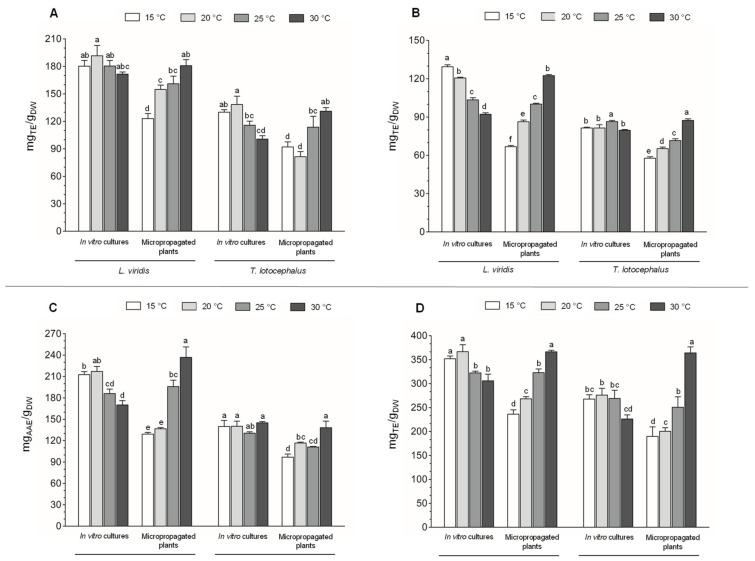
Antioxidant activity evaluated by (**A**) 2,2-diphenyl-1- picrylhydrazyl (DPPH), (**B**) 2,2-azino-bis (3-ethylbenzothiazoline-6-sulfonic acid) (ABTS), (**C**) ferric reducing antioxidant power (FRAP), and (**D**) oxygen radical absorbance capacity (ORAC) methods of the extracts from in vitro cultures and micropropagated plants of *Lavandula viridis* and *Thymus lotocephalus* exposed to different temperatures (15, 20, 25, and 30 °C). For each species, the results were analyzed by one-way analysis of variance (ANOVA) and the graph bars followed by different letters (a–f) are significantly different at *p* < 0.05 (Duncan’s New Multiple Range Test).

The protecting role of phenolic compounds against oxidative stress and lipid peroxidation is due to the inherent antioxidant activity of these compounds. The pattern of antioxidant activity is very similar between the four assays (DPPH, ABTS, FRAP, and ORAC) and between these assays and total phenolic contents (F-C and HPLC). Extracts from *L. viridis* cultures and plants demonstrated higher antioxidant capacity, especially in scavenging DPPH and ABTS radicals, as well as in reducing Fe^3+^ to Fe^2+^, than those of *T. lotocephalus*. In both species, the antioxidant activity of ex vitro plants increased with increasing temperature, and the opposite occurred in the in vitro cultures (Figure 4A–D).

Similarly, *Perilla frutescens* (Lamiaceae), an important medicinal plant, was cultivated (ex vitro) under different temperature regimes (15/10, 20/15, 25/20, 30/25, and 35/30 °C) and showed the highest DPPH scavenging capacity at 35/30 °C [20]. Also, in the Lamiaceae *M. officinalis*, ORAC assay confirmed the higher antioxidant capacity at high temperatures [27]. In contrast, *M. spicata* was subjected to 30 °C for 4 weeks and lost 21–60% of total antioxidant activity after one week and 95% in the last week [46]. *T. transcaucasicus* was exposed to 15, 20, and 25 °C and exhibited the strongest antioxidant activity at 20 °C [11]. 

### 2.4. Pearson Correlations

**Figure 5:**
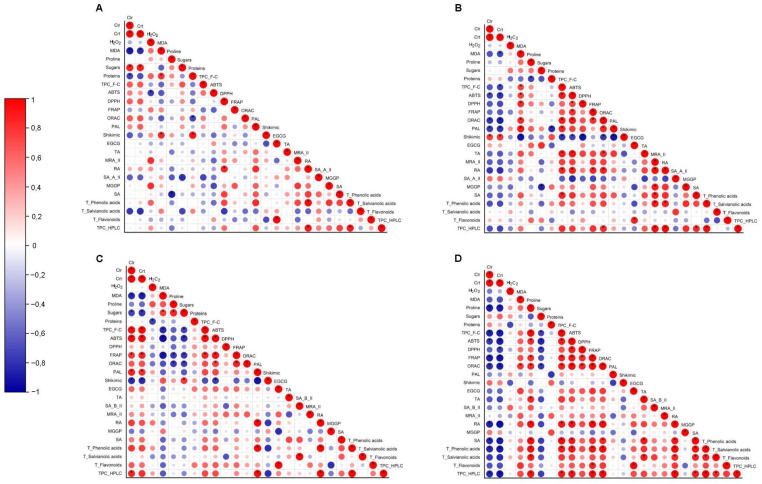
Heat map corresponding to the Pearson’s correlation coefficients (circles) between the contents photosynthetic pigments (chlorophylls and carotenoids), oxidative stress indicators (H_2_O_2_ and MDA), osmoprotectants (proline, soluble sugars, soluble proteins), shikimic/phenylpropanoid intermediates (PAL activity, shikimic acid content), total phenolic content (F-C and HPLC), total phenolic acids content (HPLC), total salvianolic acids content (HPLC), total flavonoids content (HPLC), and the most abundant individual phenolic compounds (HPLC), namely epigallocatechin gallate (EGCG), theaflavic acid (TA), salvianolic acid B isomer II (SA_B_II), methylrosmarinic acid isomer II (MRA_II), rosmarinic acid (RA), salvianolic acid A isomer II (SA_A_II), methyl 6-O-galloyl-β-D-glucopyranoside (MGGP), and sagerinic acid (SA) and antioxidant activity (DPPH, ABTS, FRAP, ORAC) from *T. lotocephalus* (**A**) in vitro cultures; (**B**) micropropagated plants; *L. viridis* (**C**) in vitro cultures; (**D**) and micropropagated plants. * Correlation is significant (*p* ≤ 0.01).

In this study, a Pearson correlation test was performed between the different parameters evaluated, namely photosynthetic pigments, oxidative stress indicators, osmoprotectants, shikimic/phenylpropanoid intermediates, total phenolic content, total phenolic acids content, total salvianolic acids content, total flavonoids content and the most abundant individual phenolic compounds from *T. lotocephalus* in vitro cultures and micropropagated plants, and *L. viridis* in vitro cultures and micropropagated plants (Figure 5).

According to the literature, the stress caused by elevated temperatures disturbs the structure and function of thylakoid membranes within the chloroplast, reducing the levels of chlorophyll [24,26]. The loss of membrane integrity under heat stress was reinforced by the Pearson correlation (Figure 5) which indicates a strong negative correlation (*p* ≤ 0.01) between lipid peroxidation and the photosynthetic pigments for both species.

The osmoprotectants, such as soluble sugars, proline, and soluble proteins, help organisms survive extreme osmotic stress and, according to some authors, their levels are highly variable and considerably improved in response to environmental stresses [16]. Soluble sugars, essential as structural and metabolic supplies for plant cells, also present an important role as osmoprotectants. In this study, this was confirmed by the Pearson correlation test in which a positive correlation was observed between total soluble sugars contents and oxidative stress indicators in *T. lotocephalus* plants and *L. viridis* cultures (Figure 5B,C). A positive correlation was also verified between these indicators and proline contents (Figure 5D) in both cultures and plants of *L. viridis*, suggesting its role in protecting cells against ROS generation and stabilizing lipid membranes. On the other hand, the Pearson test (Figure 5B–D) provides a negative correlation between total soluble protein and H_2_O_2_ content in all cases, except in *T. lotocephalus* cultures, supporting the protein damage caused by ROS. In this sense, we conclude that temperature-induced accumulation of osmolytes in cultures and plants of *L. viridis* and *T. lotocephalus* is linked with increased oxidative stress tolerance, depending on the species and/or type of plant material.

A strong correlation was observed between the total phenolic content determined by F-C assay and HPLC-HRMS (Figure 5B–D). In addition, shikimic acid and PAL enzyme are two important agents involved in the phenolic biosynthesis pathway. A strong negative correlation (Figure 5B–D) between PAL activity and shikimic acid content indicates the conversion of shikimic acid into the next intermediates for the phenylalanine synthesis. An opposite trend between PAL activity and shikimic acid content was also obtained in other plants under stress conditions [39]. A strong positive correlation was observed between PAL activity and total phenolic acids, especially in the cultures of both species (Figure 5A,C), demonstrating the important role of this enzyme in the synthesis of these compounds. Similar to results obtained by Guo et al. [13], the total flavonoids content is not in agreement with PAL activity, thus demonstrating the great complexity of the phenylpropanoid pathway. Phenolic compounds showed an important role in protecting in vitro cultures of *L. viridis* against lipid peroxidation. This evidence was validated by the strong negative correlation between total phenolic contents (by F-C and HPLC) and MDA levels (Figure 5C), evidencing a defense response of these compounds against the deleterious effects on lipid membranes caused by the higher temperatures. Specifically, salvianolic acid B (isomer II), sagerinic acid, epigallocatechin gallate, and rosmarinic acid seem to be the greatest contributors to this defense in *L. viridis* (Figure 5C). Overall, a strong positive correlation (*p* < 0.01) was obtained between antioxidant activity assays and total phenolic contents determined by HPLC and F-C methodologies (Figure 5A–D), which confirms phenolics as the major contributors to the antioxidant activities of the extracts from these species. This strong correlation was especially visible in the micropropagated plants of both species, mainly for RA, methylrosmarinic acid isomer II, sagerinic acid, and theaflavic acid (Figure 5B,D). Contrary to *T. lotocephalus*, in both cultures and plants of *L. viridis* the flavanol epigallocatechin gallate (EGCG) strongly contributed to the antioxidant capacity of the extracts. In the case of *T. lotocephalus* cultures, a strong correlation (*p* < 0.01) was observed between RA and antioxidant activity measured by DPPH assay (Figure 5A).

### 2.5. Principal Component Analysis (PCA)

**Figure 6:**
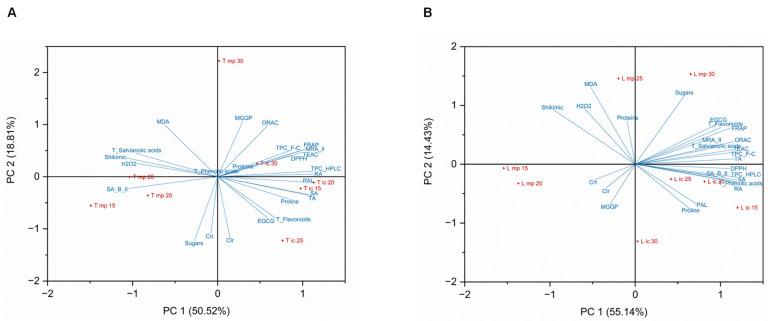
Principal component analysis (PCA) biplot of the different parameters studied, namely photosynthetic pigments (chlorophylls and carotenoids), oxidative stress indicators (H_2_O_2_ and MDA), osmoprotectants (proline, soluble sugars, soluble proteins), shikimic/phenylpropanoid intermediates (PAL activity, shikimic acid content), total phenolic content (F-C and HPLC), total phenolic acids content (HPLC), total salvianolic acids content (HPLC), total flavonoids content (HPLC), most abundant individual phenolic compounds (HPLC), namely epigallocatechin gallate (EGCG), theaflavic acid (TA), salvianolic acid B isomer II (SA_B_II), methylrosmarinic acid isomer II (MRA_II), rosmarinic acid (RA), salvianolic acid A isomer II (SA_A_II), methyl 6-O-galloyl-β-D-glucopyranoside (MGGP), sagerinic acid (SA), and antioxidant activity (DPPH, ABTS, FRAP, ORAC) in (**A**) *T. lotocephalus* in vitro cultures (T ic) and micropropagated plants (T mp), and (**B**) *L. viridis* in vitro cultures (L ic) and micropropagated plants (L mp).

To evaluate the effect of different temperatures (15, 20, 25, and 30 °C) and the influence of the micropropagation stage—in vitro cultures (ic) and ex vitro micropropagated plants (mp)—of two Lamiaceae species (*T. lotocephalus* (T) and *L. viridis* (L)), a biplot PCA was constructed with the sample scores and the variable loadings based on all parameters studied, namely photosynthetic pigments (chlorophylls and carotenoids), oxidative stress indicators (H_2_O_2_ and MDA), osmoprotectants (proline, soluble sugars, and soluble proteins), shikimic/phenylpropanoid intermediates (PAL activity and shikimic acid content), total phenolic content (determined by F-C and HPLC methods), total phenolic acids content, total salvianolic acids content, total flavonoids content, most abundant individual phenolic compounds rosmarinic acid (RA), methyl 6-O-galloyl-β-D-glucopyranoside (MGGP), epigallocatechin gallate (EGCG), sagerinic acid (SA), theaflavic acid (TA), methylrosmarinic acid isomer II (MRA_II), and salvianolic acids B isomer II (SA_B_II), and A isomer II (SA_A_II)], and antioxidant activity (determined by DPPH, ABTS, FRAP, and ORAC assays). The results for each species were separately analyzed, so two PCA biplots were obtained, one for *T. lotocephalus* (Figure 6A) and the other for *L. viridis* (Figure 6B).

The first two PC (PC1 and PC2) described 69.33% and 69.57% of the total variation in the dataset, for *T. lotocephalus* and *L. viridis*, respectively. According to the results obtained in both biplots, there is a clear separation of samples by micropropagation stage (in vitro and ex vitro). Regardless of the species studied, the samples from in vitro cultures showed higher similarities among themselves than the samples from ex vitro micropropagated plants. Furthermore, regarding the effect of the different temperatures, there is no clear trend based on growthe stage.

PCA analysis revealed a clear differentiation of micropropagated plants of both species exposed to extreme temperatures (15 and 30 °C). Moreover, in these samples, at the highest temperature, a great distance was observed between chlorophyll/proteins and H_2_O_2_ contents, and in contrast a great proximity between MDA and H_2_O_2_ contents. This was also supported by the negative correlation between the contents of H_2_O_2_ and chlorophyll/proteins and by the positive correlation between MDA and H_2_O_2_ (Figure 5A–D). These results could indicate an accelerated process of cellular aging (leaf senescence) at high temperatures, characterized by damage in proteins, membrane lipids, and chlorophylls caused by ROS [17,35]. On the other hand, the PCA analysis suggests that, in both species, the lowest temperatures (15 and 20 °C) promote the production of phenolics and flavonoids, including of the major phenolic compound (RA), in in vitro cultures, and the shikimic acid in micropropagated plants. In the case of in vitro cultures of *L. viridis*, the lowest temperatures also improve antioxidant activity.

## 3. Materials and Methods

### 3.1. Chemicals and Reagents

L-Proline (>85%), ninhydrin, choline chloride, and (±)-6-hydroxy-2,5,7,8-tetramethylchromane-2-carboxylic acid (Trolox) were purchased from Acros Organics (Geel, Germany). Lactic acid was acquired from Panreac (Barcelona, Spain), and gallic acid, Folin–Ciocalteu (F-C) reagent, and sodium carbonate from VWR (Leuven, Belgium). Sodium hydroxide (NaOH), ascorbic acid, potassium iodide, and acetic acid were supplied by Merck (Darmstadt, Germany). Trichloroacetic acid (TCA), 2-thiobarbituric acid (TBA), hydrogen peroxide (H_2_O_2_), L-proline (≥99%), indole-3-acetic acid, toluene, Bradford reagent, glucose, shikimic acid, periodic acid, cinnamic acid, Bovine Serum Albumin (BSA), luteolin, protocatechuic acid, and epigallocatechin gallate, HPLC-MS-grade water, HPLC-MS grade acetonitrile, and formic acid were purchased from Sigma–Aldrich (Steinheim, Germany). Quercetin and rosmarinic acid were acquired from Extrasynthese (Genay, France). Catechin, caffeic, and ρ-coumaric acids were supplied by AASC Ltd. (Southhampton, UK). Phenol, glycine, sulfuric acid, 6-benzyladenine were acquired from Fluka (Buchs, Switzerland), and L-phenylalanine was obtained from Fisher Scientific (Leicestershire, UK).

### 3.2. Micropropagation and Temperature Experiments

#### 3.2.1. Culture Conditions and Micropropagation

In vitro cultures of *T. lotocephalus* and *L. viridis* were multiplied in MS medium [49,50] and half strength MS medium supplemented with 0.2 mg/L of 6-benzyladenine (BA) [51], respectively. Media, containing 2% (*w*/*v*) sucrose and 0.7% (*w*/*v*) agar, were autoclaved at 121 °C for 20 min. Cultures (7 shoots for each Erlenmeyer flasks) were incubated at a temperature of 25 ± 1 °C, with 16/8 h (light/dark) of photoperiod. To produce micropropagated plants, in vitro shoots of *T. lotocephalus* and *L. viridis* were inoculated in ¼ MS medium supplemented with 0.5 mg/L indole-3-acetic acid (IAA) and MS medium, respectively, for 8 weeks, for rooting. Plantlets with well-developed roots were transplanted to plastic pots (350 mL) containing a peat: vermiculite (3:1, *v*/*v*) mixture. The plantlets were acclimatized to ex vitro conditions for 4–6 weeks in transparent polyethylene boxes at 25 ± 1 °C, with 16/8 h (light/dark) of photoperiod. The boxes were gradually opened to expose the plantlets to progressively reduced relative humidity. Plantlets were frequently watered with distilled water and with a nutritive solution (MS liquid medium). 

#### 3.2.2. Temperature Experiments

**Figure 7:**
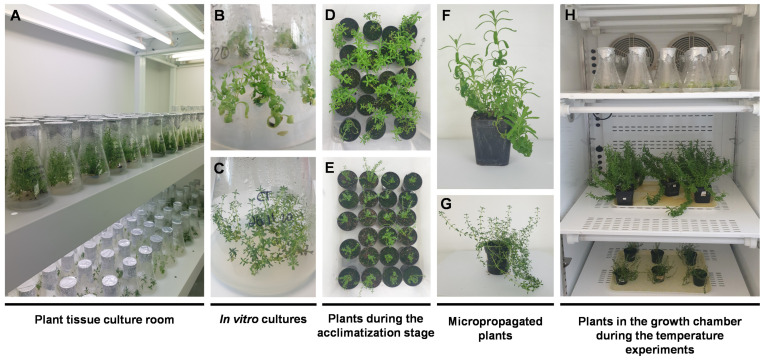
Plants at different stages of the micropropagation process used in the temperature experiments. (**A**) plant tissue culture room; (**B**) in vitro cultures of *L. viridis* (**C**) and *T. lotocephalus*; (**D**) plants of *L. viridis* and (**E**) *T. lotocephalus* under acclimatization phase; (**F**) micropropagated plants of *L. viridis* and (**G**) *T. lotocephalus*; (**H**) in vitro cultures and micropropagated plants of both species during the temperature experiments in a growth chamber under controlled conditions.

In vitro cultures growing in multiplication media for 7 weeks and micropropagated plants growing in ex vitro conditions for 12 months were used in temperature experiments. Cultures and plants of both species were maintained for 2 weeks in a growth chamber (750 E, Aralab, Lisbon, Portugal) equipped with four Osram L 18 W/840 lamps (16/8 h, light/dark) at a constant temperature of 15, 20, 25, or 30 °C (±0.5 °C), with 50–60% air humidity. After each temperature treatment, part of the plant material (in vitro cultures and aerial parts of micropropagated plants) was oven-dried (at 40 °C) to produce plant extracts for phenolic profile evaluation and the remainder was frozen in liquid nitrogen and kept at –80 °C until use. To test the effect of each temperature, 12 Erlenmeyer flasks with in vitro cultures and 5 micropropagated plants were used. Figure 7 shows the different stages of plant growth (in vitro cultures and micropropagated plants) used in the temperature experiments. 

### 3.3. Photosynthetic Pigments Determination

Photosynthetic pigments (chlorophylls and carotenoids) were extracted according to the Lichtenthaler [52] protocol. For that, a maceration was performed using fresh plant material (25 mg) and pure acetone (4 mL). The optical density was measured at 470, 644.8, and 661.6 nm.

### 3.4. Oxidative Stress Markers

Hydrogen peroxide (H_2_O_2_) content was calculated following a protocol from Loreto and Velikova [53] with minor modifications. Concisely, fresh plant material (0.1 g) was completely homogenized with 1 mL of 0.1% (*w*/*v*) TCA. The extract was centrifuged (15 min, 12,000× g) and the supernatant was collected and mixed with 1 M potassium iodide (0.4 mL) and 10 mM potassium phosphate buffer (0.2 mL). After 30 min in the dark, the optical density was measured at 390 nm. The results were determined as micromoles of H_2_O_2_ equivalents per gram of fresh weight (µmol_H2O2_/g_FW_).

The method described by Hodges et al. [54], with slight modifications, was employed to determine malondialdehyde (MDA) content as an estimation of lipid peroxidation. The supernatant was the same used in the H_2_O_2_ content protocol. The diluted supernatant (0.5 mL) was thoroughly mixed with 0.5% (*w*/*v*) TBA in 20% (*w*/*v*) TCA and 20% (*w*/*v*) TCA, heated at 95 °C for 30 min, and then cooled on ice. The optical density of the mixture was measured at 440, 532, and 660 nm. The results were estimated as nanomoles of MDA equivalents per gram of fresh weight (nmol_MDA_/g_FW_).

### 3.5. Osmoprotectants Determination

To quantify the amino acid proline, a triple extraction (at 80 °C for 30 min) was performed using 0.25 g of plant material and 0.5 mL of 80% (*v*/*v*) ethanol, according to Martins et al. [55]. After incubation for 1 h at 100 °C with 1% (*w*/*v*) ninhydrin reagent previously prepared in 60% (*v*/*v*) acetic acid, 1 mL toluene was added. The optical density of the organic phase was measured at 520 nm. The results were expressed as micromoles of proline equivalents per gram of fresh weight (µmol_PRO_/g_FW_).

Total soluble proteins were quantified using the Bradford method [56] and Bovine Serum Albumin (BSA) as a protein standard. For that, 250 µL of Bradford reagent were mixed with 5 µL of plant extract. After 30 min at room temperature, the optical density was determined at 595 nm.

The content of soluble sugars was determined by the phenol sulfuric acid method, according to Abid et al. [57], with slight alterations. Plant material (0.025 g) was extracted in 2 mL of 80% (*v*/*v*) ethanol in a water bath at 80 °C for 30 min. The supernatant (0.1 mL) was mixed with the same amount of distilled water, 9% (*w*/*v*) phenol (0.2 mL), and sulfuric acid (96%) (1 mL). After incubation at room temperature for 30 min, the optical density was measured at 490 nm. Glucose was used as a standard and the results were estimated as milligrams of glucose equivalents per gram of fresh weight (mg_GLU_/g_FW_).

### 3.6. Secondary Metabolism Keys

#### 3.6.1. Shikimic Acid Content

The shikimic acid content was determined using the Alzandi and Naguib [39] procedure. Fresh plant material (0.1 g) was squeezed in 0.25 M HCl (2 mL) and centrifuged (20,000× g, 15 min, 4 °C). Fifty microliters of the supernatant were mixed with 1% (*w*/*v*) periodic acid (0.5 mL) and incubated for 3 h at room temperature. After this period, 1 M NaOH (0.5 mL) and 0.1 M fresh glycine (0.3 mL) were added to the reaction. The optical density was recorded at 380 nm. The results were estimated as milligrams of shikimic acid equivalents per gram of fresh weight (mg_SA_/g_FW_).

#### 3.6.2. Phenylalanine Ammonia Lyase (PAL) Activity

PAL activity was measured as described by Zhan et al. [58], wherein 50 mg of fresh plant material was homogenized in 1000 µL phosphate buffer (Na e K, 50 mM, pH 8.0) and centrifuged (20,000× g, 20 min, 4 °C). Five hundred microliters of L-phenylalanine (50 mM) were mixed with 100 µL of supernatant and 1400 µL of the buffer. The mixture was incubated at 37 °C for 1 h. The optical density was measured at 290 nm and the enzyme activity was expressed as μmol cinnamic acid per hour, per milligram of protein (μmol_CA_/mg_protein_/h). The protein content of enzyme extracts was evaluated using the Bradford [56] method.

#### 3.6.3. Phenolic Compounds Extraction and Quantification

##### Ultrasound-Assisted Extraction

The plant material, dried at 40 °C and ground until <2 mm, was extracted with a natural deep eutectic solvent, choline chloride: lactic acid (1:2) with 30% (*w*/*w*) distilled water, given the advantages of these eco-friendly solvents for the extraction of phenolics from Lamiaceae plants [4,59]. A plant/solvent ratio of 2.5:100 (*w*/*v*) and an ultrasound-assisted extraction for 30 min at 50 °C were used according to Mansinhos et al. [59]. At the final stage, the extracts were filtered and kept at −20 °C until use.

##### Phenolics Quantification

The spectrophotometric analysis of total phenolic content (TPC) was performed according to Ainsworth and Gillespie [60]. A mixture containing 0.2 mL F-C reagent (10%, *v*/*v*), 0.1 mL extract, and 0.8 mL sodium carbonate (700 mM) was incubated for 2 h. Gallic acid was used as a reference and the optical density was determined at 765 nm. The results were expressed as milligrams of gallic acid equivalents per gram of dry weight (mg_GAE_/g_DW_).

To analyze the phenolic profile of the extracts, the chromatographic technique HPLC-HRMS was used according to Mansinhos et al. [4] and conducted on a Dionex Ultimate 3000 HPLC system with an HPLC pump and an autosampler operating at 10 °C (Thermo Scientific, San Jose, CA, USA); the sample separation was accomplished on a Kinetex column (150 × 4.6 mm i.d. 5 μm 100 A C18) (Phenomenex, UK), at 40 °C, flowing at a rate of 1 mL/min. The solvent system contained distilled water (solvent A) and acetonitrile (solvent B), both with formic acid (0.1%). The gradient mode was 0 min—90% A; 10 min—74% A; 22 min—35% A; 30 min—5% A; 40 min—5% A; 40.1 min—90% A; and 45 min—90% A. The column flow rate (0.2 mL/min) was directed to an Exactive Orbitrap mass spectrometer (Thermo Fisher Scientific, San José, CA, USA) supplied with a heated electrospray ionization probe (HESI). Negative ions were analyzed at scan mode of auto MS/MS (100–1000 m/z). Full scans were registered with a resolution of 50,000 and with full automatic gain control (AGC) target of 1,000,000 charges and using 2 microscans. The analyses were as well based on in-source collision-induced dissociation scans (25 eV) with a spray voltage of 4000 V, heater temperature of 150 °C, the capillary temperature of 320 °C, the flow rates of sheath gas of 25 units, and the auxiliary gas of 5 units. Xcalibur (Thermo Fisher Scientific, San José, CA, USA) was the software used for data acquisition and processing. Every week, the Exactive Orbitrap was externally calibrated using ready-to-use calibration mixtures of Pierce ESI Negative Ion Calibration Solution and Pierce LQT ESI Positive Ion Calibration Solution (Thermo Fisher Scientific, San José, CA, USA). A quality control (QC) sample, composed of identical aliquots of a representative pool of the samples (plant extracts), was applied to assess and ensure that the analytical process was performed appropriately. The QC sample was injected regularly throughout the run and was used to monitor drifts and to determine the variance of a metabolite feature (below 20%). The compounds were identified according to the retention time and the exact mass in combination with standards. In the absence of the standards, the compound was tentatively identified by comparing the theoretical exact mass of the molecular ion with the defined accurate mass of the molecular ion, and then searched for in several metabolite databases, namely PubChem, Phenol Explorer, Metlin, and ChemSpider. The identification of the compounds was performed following the MSI MS levels [61]. Supplementary Table S1 sum up the chemical formula, theoretical exact mass, delta ppm, retention time (RT), and MSI MI levels of the compounds. Limits of detection (LOD) and quantification (LOQ) ranged from 0.10 to 228.13 µg/L and 0.33 to 760.43 µg/L, respectively. The assumptions used to quantify the phenolic compounds are summarized in Supplementary Table S2 and the results, expressed in milligrams per kilogram of dry weight (mg/kg_DW_) or grams per kilogram of dry weight (g/kg_DW_), are detailed in Supplementary Tables S3 and S4.

##### Antioxidant Activity

The antioxidant capacity of the extracts was assessed according to Mansinhos et al. [59], using four different methods: FRAP (a single electron transfer-based assay), ORAC (an atom hydrogen transfer-based assay), and ABTS and DPPH (a mixture of the two previously mentioned mechanisms). Results were expressed as milligrams of ascorbic acid (FRAP) or Trolox (ABTS, DPPH, ORAC) equivalents per gram of dry weight (mg_AAE_/g_DW_ or mg_TE_/g_DW_).

### 3.7. Statistical Analysis

The present results, described as mean ± standard error, were analyzed by one-way analysis of variance (ANOVA) and Duncan’s New Multiple Range Test (*p* < 0.05) using the statistical software IBM SPSS Statistics for Windows (version 26.0, Armonk, NY: IBM Corp). Principal component analysis (PCA) and the Pearson correlation (*p* ≤ 0.01) were analyzed by the software OriginPro, version 2022 (OriginLab Corporation, Northampton, MA, USA).

## 4. Conclusions

This first report studying the effect of temperature on in vitro cultures and micropropagated plants of *L. viridis* and *T. lotocephalus* demonstrated the considerable impact of the extreme temperatures (15 and 30 °C) on both species, namely at physiological, biochemical, metabolic, and biological levels. Osmoprotectants and phenolic compounds demonstrated their important role in the response of both Lamiaceae species to temperature stress, proving to be suitable for the comprehension of *L. viridis* and *T. lotocephalus* stress responses. However, considering the climate change context, a deeper analysis of the effects of more extreme temperatures is needed to understand the possible effects of elevated temperatures on the primary and secondary metabolism of these two Mediterranean plants. Even so, temperature showed to be a great abiotic elicitor to produce RA, especially under in vitro conditions. Particularly, in vitro cultures of *L. viridis* exposed for two weeks to 15 °C demonstrated to be a simple and a very advantageous method to produce higher amounts of this valuable bioactive compound.

## Figures and Tables

**Table 1:** Effect of exposure to different temperatures (15, 20, 25, and 30 °C) on Cltotal, Crt, MDA, H_2_O_2_, soluble sugar, soluble proteins, and proline contents of in vitro cultures and micropropagated plants of *Lavandula viridis* and *Thymus lotocephalus*.

Treatment	Cltotal (mg/g_FW_)	Crt (mg/g_FW_)	MDA (nmol/g_FW_)	H_2_O_2_ (µmol/g_FW_)	Soluble Sugars (mg/g_FW_)	Soluble Proteins (mg/g_FW_)	Proline (µmol/g_FW_)
*L. viridis*							
In vitro cultures							
15 °C	1.34 ± 0.06 a	0.30 ± 0.01 a	23.7 ± 1.42 e	2.83 ± 0.11 e	31.7 ± 0.20 de	7.92 ± 0.06 b	0.99 ± 0.09 b
20 °C	0.88 ± 0.02 c	0.22 ± 0.01 b	25.5 ± 1.35 ef	1.10 ± 0.13 f	28.6 ± 1.53 e	9.90 ± 0.13 a	0.31 ± 0.03 d
25 °C	0.61 ± 0.02 d	0.14 ± 0.00 c	32.6 ± 1.99 de	2.19 ± 0.06 e	64.5 ± 1.22 a	9.97 ± 0.05 a	1.53 ± 0.11 a
30 °C	0.35 ± 0.01 e	0.09 ± 0.00 d	35.6 ± 2.10 cd	2.77 ± 0.14 e	54.1 ± 0.85 b	6.10 ± 0.33 c	1.43 ± 0.08 a
Micropropagated plants							
15 °C	1.01 ± 0.04 b	0.29 ± 0.02 a	45.6 ± 2.58 b	9.63 ± 0.18 a	50.6 ± 0.63 b	8.33 ± 0.14 b	0.11 ± 0.01 de
20 °C	1.31 ± 0.07 a	0.30 ± 0.01 a	42.3 ± 2.34 bc	3.80 ± 0.21 d	37.6 ± 0.97 c	9.65 ± 0.07 a	0.08 ± 0.01 e
25 °C	0.86 ± 0.04 c	0.21 ± 0.01 b	58.8 ± 6.42 a	5.77 ± 0.37 c	35.6± 2.82 cd	10.2 ± 0.05 a	0.23 ± 0.08 de
30 °C	0.25 ± 0.00 e	0.10 ± 0.00 d	56.9 ± 3.00 a	8.44 ± 0.47 b	32.8 ± 1.35 de	8.31 ± 0.55 b	0.53 ± 0.04 c
*T. lotocephalus*							
In vitro cultures							
15 °C	1.23 ± 0.02 a	0.32 ± 0.01 a	18.6 ± 0.54 e	1.16 ± 0.05 d	50.7 ± 0.58 a	7.92 ± 0.57 b	6.05 ± 0.31 a
20 °C	1.03 ± 0.02 c	0.27 ± 0.01 b	16.9 ± 0.47 d	0.93 ± 0.15 d	36.0 ± 0.81 cd	6.52 ± 0.18 c	1.18 ± 0.24 d
25 °C	1.17 ± 0.03 ab	0.26 ± 0.00 b	11.7 ± 0.51 e	0.69 ± 0.15 d	42.6 ± 1.49 b	6.45 ± 0.22 c	3.76 ± 0.21 c
30 °C	0.44 ± 0.03 e	0.12 ± 0.00 c	30.9 ± 2.02 b	1.13 ± 0.06 d	28.9 ± 0.12 e	10.1 ± 0.81 a	4.51 ± 0.18 b
Micropropagated plants							
15 °C	0.78 ± 0.00 d	0.24 ± 0.01 b	30.5 ± 2.17 b	7.99 ± 0.66 a	42.3 ± 1.28 b	6.31 ± 0.47 c	n.d.
20 °C	1.12 ± 0.06 bc	0.31 ± 0.01 a	24.7 ± 1.84 c	2.24 ± 0.22 c	33.5 ± 0.35 d	8.00 ± 0.40 b	n.d.
25 °C	1.01 ± 0.05 c	0.27 ± 0.01 b	41.2 ± 1.77 a	5.41 ± 0.48 b	49.9 ± 1.14 a	5.81 ± 0.23 c	0.05 ± 0.01 e
30 °C	0.23 ± 0.03 f	0.09 ± 0.00 d	44.5 ± 0.78 a	4.72 ± 0.24 b	38.5 ± 0.39 c	6.38 ± 0.44 c	n.d.

For each variable and species, the results were analyzed by one-way analysis of variance (ANOVA) and the values followed by different letters (a to f) are significantly different at *p* < 0.05 (Duncan’s New Multiple Range Test). Cltotal: total chlorophyll; Crt: carotenoids; MDA: malondialdehyde; H_2_O_2_: hydrogen peroxide; and n.d.: not defined.

## Data Availability

The data presented are included within the article and in the Supplementary Materials.

## Supplementary Materials

The following supporting information can be downloaded at: https://www.mdpi.com/article/10.3390/plants11243516/s1, Table S1: HPLC-HRMS data of identified phenolics in *Thymus lotocephalus* and *Lavandula viridis* extracts, Table S2: Summary of HPLC-HRMS criterion for quantification of phenolics in *Thymus lotocephalus* and *Lavandula viridis* extracts, Table S3: Qualitative and quantitative [(mg/kg_DW_ or g/kg_DW_ (marked with *), mean ± SE)] analysis by HPLC-HRMS of the phenolic profile from in vitro cultures and micropropagated plants of *Lavandula viridis* exposed to different temperatures (30, 25, 20, and 15 °C) for two weeks, Table S4: Qualitative and quantitative [(mg/kg_DW_ or g/kg_DW_ (marked with *), mean ± SE)] analysis by HPLC-HRMS of the phenolic profile from in vitro cultures and micropropagated plants of *Thymus lotocephalus* exposed to different temperatures (30, 25, 20, and 15 °C) for two weeks.
