# Supplementary material for: Impact of Temperature on Phenolic and Osmolyte Contents in In Vitro Cultures and Micropropagated Plants of Two Mediterranean Plant Species, Lavandula viridis and Thymus lotocephalus
Source: Plants (Basel). 2022 Dec 14;11(24):3516. doi: 10.3390/plants11243516 (PMC9787929; doi:10.3390/plants11243516)
Supplement: Supplementary file 1 [file plants-11-03516-s001.zip › plants-2060126-supplementary.pdf]

Impact of Temperature on Phenolic and Osmolyte Contents in in Vitro Cultures and Micropropagated Plants of Two Mediterranean Plant Species, *Lavandula viridis* and *Thymus lotocephalus*

Inês Mansinhos [1], Sandra Gonçalves [1,*], Raquel Rodríguez-Solana [1,2], José Luis Ordóñez-Díaz [2], José Manuel Moreno-Rojas [2,3] and Anabela Romano [1,*]

[1] MED—Mediterranean Institute for Agriculture, Environment and Development & CHANGE—Global Change and Sustainability Institute, Faculdade de Ciências e Tecnologia, Universidade do Algarve, Campus de Gambelas, 8005-139 Faro, Portugal

[2] Department of Agroindustry and Food Quality, Andalusian Institute of Agricultural and Fisheries Research and Training (IFAPA), Avenida Menendez-Pidal, SN, 14004 Córdoba, Spain

[3] Foods for Health Group, Instituto Maimónides de Investigación Biomédica de Córdoba (IMIBIC), Avenida Menendez-Pidal, SN, 14004 Córdoba, Spain

* Correspondence: smgoncalves@ualg.pt (S.G.); aromano@ualg.pt (A.R.); Tel.: +351-289800910 (A.R.)

Supplementary information

Table S1. HPLC-HRMS data of identified phenolics in *Thymus lotocephalus* and *Lavandula viridis* extracts.

Compound identity	Chemical formula	Theoretical exact mass $[M-H]^-$ *(m/z)*	Delta ppm (error)	RT (min)	MSI MI level*
Phenolic acids					
Salvianolic acid A isomer I [1]	$C_{26}H_{22}O_{10}$	493.1129	0.30	7.16	2
Salvianolic acid A isomer II [1]	$C_{26}H_{22}O_{10}$	493.1129	-0.76	10.6	2
Salvianolic acid A isomer III [1]	$C_{26}H_{22}O_{10}$	493.1129	0.36	11.47	2
Salvianolic acid A isomer IV [1]	$C_{26}H_{22}O_{10}$	493.1129	-0.82	13.1	2
Salvianolic acid B /Salvianolic acid L isomer I [1]	$C_{36}H_{30}O_{16}$	717.1456	-2.62	9.22	2
Salvianolic acid B /Salvianolic acid L isomer II [1]	$C_{36}H_{30}O_{16}$	717.1456	-0.58	11.63	2
Salvianolic acid B /Salvianolic acid L isomer III [1]	$C_{36}H_{30}O_{16}$	717.1456	-0.59	13.16	2
Salvianolic acid B / Salvianolic acid L isomer IV [1]	$C_{36}H_{30}O_{16}$	717.1456	-0.50	14.34	2
Salvianolic acid C [1]	$C_{18}H_{17}O_{9}$	377.0873	-0.74	5.37	2
Salvianolic acid F isomer I [1]	$C_{17}H_{13}O_{6}$	313.0712	1.61	14.6	2
Salvianolic acid F isomer II[1]	$C_{17}H_{13}O_{6}$	313.0712	0.15	15.26	2
Salvianolic acid I / Melitric acid I isomer I [1]	$C_{27}H_{22}O_{12}$	537.1034	0.63	6.54	2
Salvianolic acid I / Melitric acid I isomer II [1]	$C_{27}H_{22}O_{12}$	537.1034	-0.28	7.16	2
Salvianolic acid I / Melitric acid I isomer III [1]	$C_{27}H_{22}O_{12}$	537.1034	-0.73	11.55	2
Caffeic acid	$C_{9}H_{8}O_{4}$	179.0338	-0.14	4.81	1
Fertaric acid [2]	$C_{14}H_{14}O_{9}$	325.0557	0.68	4.71	2
Melitric acid B isomer I [1]	$C_{27}H_{20}O_{11}$	519.0922	0.66	11.69	2
Melitric acid B isómer II [1]	$C_{27}H_{20}O_{11}$	519.0922	-0.63	14.3	2

Methyl 6-O-galloyl-β-D-glucopyranoside [3]	$C_{14}H_{18}O_{10}$	345.0793	-5.81	2.64	2
Methylrosmarinic acid isomer I [1]	$C_{19}H_{18}O_8$	373.0921	1.14	12.56	2
Methylrosmarinic acid isomer II [1]	$C_{19}H_{18}O_8$	373.0921	0.98	13.97	2
Rosmarinic acid	$C_{18}H_{16}O_8$	359.0761	0.77	10	1
Sagerinic acid [1]	$C_{36}H_{32}O_{16}$	719.1612	-1.21	10.07	2
Salviaflaside [1]	$C_{24}H_{26}O_{13}$	521.1288	0.12	8.1	2
Flavonoids					
Dihydromorelloflavone [4]	$C_{30}H_{22}O_{11}$	557.1083	0.13	5.37	2
Epigallocatechin gallate	$C_{22}H_{18}O_{11}$	457.0765	0.27	5.34	1
Luteolin	$C_{15}H_{10}O_6$	285.0393	0.55	13.97	1
Luteolin-7-O-glucuronide [5]	$C_{21}H_{18}O_{12}$	461.0714	-0.03	8.67	2
Theaflavic acid [6]	$C_{21}H_{16}O_{10}$	427.0636	-5.28	10.04	2
Couramin derivatives					
Herniarin [7]	$C_{10}H_8O_3$	175.0389	-1.83	12.51	2
Hydroxybenzaldehydes					
Protocatechuic aldehyde [8]	$C_7H_6O_3$	137.0239	0.07	3.96	2

*Metabolite standards initiative metabolite identification (MSI MI) levels. Reference standards were available for all compounds identified at MSI MI level 1. Results expressed as equivalents of rosmarinic acid (1), caffeic acid (2), gallic acid (3), quercetin (4), luteolin (5), catechin (6), *p* -coumaric acid (7), protocatechuic acid (8).

Table S2. Summary of HPLC-HRMS criterion for quantification of phenolics in *Thymus lotocephalus* and *Lavandula viridis* extracts.

Compound	Linear range (mg/L)	Intercept	Slope	R^2	LOD (μg/L)	LOQ (μg/L)
Epigallocatechin gallate	3.12-50	-69150	21667	0.9976	228.13	760.46
Caffeic acid	0.05-8	209431	5791726	0.9999	0.95	3.16
Protocatechuic acid	0.05-10	150321	2058784	0.9995	3.92	13.06
Gallic acid	0.1-50	124985	3119019	0.9993	1.38	4.60
p -Coumaric acid	0.5-6	2090	84084	0.9979	43.91	146.38
Luteolin	0.03-91	7840120	-158687	0.9997	0.60	2.00
Quercetin	0.1-3.12	-1347970	9918400	0.9975	0.10	0.33
Catechin	0.8-25	-632768	2024864	0.9994	3.25	10.82
Rosmarinic acid	0.10-80	-2568152	1567304	0.9917	30.11	100.38

Table S3. Qualitative and quantitative [(mg/kg$_{DW}$ or g/kg$_{DW}$ (marked with *), mean ± SE)] analysis by HPLC-HRMS of the phenolic profile from *in vitro* cultures and micropropagated plants of *Lavandula viridis* exposed to different temperatures (30, 25, 20, and 15 °C) for two weeks.

Compound	Treatment							
	In vitro cultures				*Micropropagated plants*			
	30 °C	25 °C	20 °C	15 °C	30 °C	25 °C	20 °C	15 °C
Phenolic acids								
Salvianolic acid A isomer I	<LOQ	<LOQ	<LOQ	<LOQ	<LOQ	<LOQ	<LOQ	<LOQ
Salvianolic acid A isomer II	194 ± 4 bc	282 ± 34 bc	470 ± 121 a	283 ± 32 bc	347 ± 58 ab	177 ± 39 bc	110 ± 17 c	155 ± 31 c
Salvianolic acid A isomer III	<LOQ	<LOQ	<LOQ	<LOQ	<LOQ	<LOQ	<LOQ	<LOQ
Salvianolic acid A isomer IV	n.d.	n.d.	n.d.	n.d.	n.d.	n.d.	<LOQ	<LOQ
Salvianolic acid B /Salvianolic acid L isomer I	n.d.	n.d.	n.d.	n.d.	n.d.	<LOQ	<LOD	n.d.
*Salvianolic acid B /Salvianolic acid L isomer II	4.28 ± 0.30 a	4.03 ± 1.11 a	5.73 ± 1.76 a	4.94 ± 0.85 a	4.17 ± 0.31 a	4.11 ± 0.85 a	2.73 ± 0.47 a	3.91 ± 0.21 a
Salvianolic acid B /Salvianolic acid L isomer III	147 ± 13 c	<LOD	<LOQ	<LOQ	371 ± 9 a	235 ± 15 b	<LOQ	127 ± 6 c
Salvianolic acid B / Salvianolic acid L isomer IV	n.d.	<LOD	<LOD	<LOD	<LOQ	133 ± 12	127 ± 9	<LOQ
Salvianolic acid C	<LOQ	<LOQ	<LOQ	<LOQ	<LOQ	<LOQ	<LOQ	<LOQ
Salvianolic acid F isomer I	n.d.	n.d.	n.d.	n.d.	n.d.	n.d.	n.d.	n.d.
Salvianolic acid F isomer II	401 ± 9 a	254 ± 48 abcd	339 ± 63 ab	306 ± 56 abc	231 ± 79 bcd	181 ± 5 cd	131 ± 4 d	139 ± 2 d
Salvianolic acid I / Melitric acid A isomer I	403 ± 85 b	271 ± 41 b	187 ± 8 b	<LOQ	1015 ± 169 a	306 ± 49 b	387 ± 88 b	444 ± 79 b
Salvianolic acid I / Melitric acid A isomer II	<LOD	170 ± 19	152 ± 10	<LOQ	224 ± 38	<LOQ	<LOQ	<LOQ
Salvianolic acid I / Melitric acid A isomer III	<LOD	<LOD	<LOD	<LOD	<LOD	<LOD	<LOD	<LOD
****Total salvianolic acids***	5.43 ± 0.25 a	5.01 ± 0.96 a	6.88 ± 1.96 a	5.53 ± 0.94 a	6.36 ± 0.66 a	5.14 ± 0.96 a	3.48 ± 0.55 a	4.77 ± 0.10 a
Caffeic acid	144 ± 6 cd	108 ± 17 d	109 ± 31 d	128 ± 7 cd	182 ± 1 abc	219 ± 22 a	202 ± 12 ab	149 ± 7 bcd
Fertaric acid	65 ± 3 c	10 ± 4 c	<LOQ	<LOQ	275 ± 0 a	164 ± 3 b	199 ± 52 ab	260 ± 7 a
Melitric acid B isomer I	202 ± 22 a	190 ± 71 a	260 ± 64 a	226 ± 24 a	184 ± 1 a	189 ± 33 a	128 ± 18 a	148 ± 15 a
Melitric acid B isomer II	n.d.	n.d.	n.d.	n.d.	<LOD	<LOD	<LOD	<LOD
*Methyl 6-O-galloyl-β-D-glucopyranoside	6.60 ± 0.07 a	5.98 ± 0.61 a	6.29 ± 0.08 a	5.79 ± 0.13 a	4.39 ± 2.14 a	6.71 ± 0.92 a	6.53 ± 0.13 a	5.58 ± 0.53 a

Methylrosmarinic acid isomer I	277 ± 28 ab	274 ± 51 ab	371 ± 115 ab	435 ± 139 a	213 ± 15 ab	418 ± 27 a	172 ± 29 b	151 ± 7 b
*Methylrosmarinic acid isomer II	0.94 ± 0.02 ab	1.07 ± 0.20 ab	1.43 ± 0.43 ab	1.37 ± 0.32 ab	1.04 ± 0.01 ab	1.50 ± 0.03 a	0.96 ± 0.14 ab	0.72 ± 0.03 b
*Rosmarinic acid	71.9 ± 0.51 b	70.1 ± 0.64 bc	67.3 ± 1.01 bc	92.6 ± 4.74 a	64.9 ± 0.76 c	48.2 ± 2.04 d	17.1 ± 1.22 e	15.9 ± 0.83 e
*Sagerinic acid	5.67 ± 0.66 ab	5.05 ± 0.62 abc	6.51 ± 2.36 ab	7.87 ± 1.39 a	6.18 ± 1.38 ab	2.88 ± 0.82 bcd	1.11 ± 0.21 cd	0.52 ± 0.11 d
Salviaflaside	185 ± 2 a	<LOQ	134 ± 27 a	153 ± 34 a	153 ± 26 a	126 ± 27 a	109 ± 44 a	185 ± 5 a
***Total phenolic acids**	91.4 ± 0.53 b	87.7 ± 0.61 b	89.3 ± 2.91 b	114 ± 6.80 a	83.8 ± 3.42 b	65.5 ± 4.73 c	30.0 ± 1.65 d	28.4 ± 1.36 d
Flavonoids								
Dihydromorelloflavone	319 ± 27 a	267 ± 26 ab	226 ± 45 ab	280 ± 9 ab	255 ± 66 ab	249 ± 33 ab	185 ± 7 b	238 ± 43 ab
*Epigallocatechin gallate	4.27 ± 0.21 a	9.97 ± 1.91 a	7.99 ± 2.81 a	10.5 ± 0.05 a	9.39 ± 3.45 a	9.45 ± 2.27 a	4.45 ± 1.32 a	4.32 ± 1.22 a
Luteolin	n.d.	n.d.	n.d.	n.d.	n.d.	n.d.	n.d.	14 ± 1
Luteolin-7-O-glucuronide	<LOQ	12 ± 1 b	12 ± 1 b	18 ± 0 a	12 ± 0 b	<LOQ	<LOQ	16 ± 1 a
*Theaflavic acid	1.29 ± 0.01 a	1.62 ± 0.30 a	1.39 ± 0.47 a	1.58 ± 0.26 a	1.57 ± 0.43 a	1.18 ± 0.32 a	0.89 ± 0.04 a	0.78 ± 0.14 a
Total flavonoids	5.88 ± 0.20 c	11.9 ± 1.58 a	9.62 ± 2.30 ab	12.4 ± 0.31 a	11.2 ± 2.95 ab	10.9 ± 1.92 ab	5.53 ± 1.28 c	5.36 ± 1.04 c
Coumarin derivative								
Herniarin	206 ± 18 a	<LOQ	204 ± 54 a	265 ± 96 a	<LOQ	277 ± 7 a	<LOQ	<LOQ
Hydroxybenzaldehyde								
Protocatechuic aldehyde	272 ± 4 ab	156 ± 23 cd	133 ± 48 d	167 ± 11 bcd	232 ± 62 abcd	284 ± 6 a	254 ± 11 abc	232 ± 12 abcd
***Total phenolic compounds**	97.8 ± 0.75 b	99.8 ± 2.16 b	99.3 ± 0.72 b	126 ± 7.19 a	95.3 ± 0.53 b	77.0 ± 2.83 c	35.8 ± 2.91 d	34.0 ± 0.31 d

Notes: n.d. – not detected; LOD – limit of detection; LOQ – limit of quantification. The results were analyzed using a one-way analysis of variance (ANOVA) followed by Duncan's New Multiple Range Test. Distinct letters (a to e) in each row and for each phenolic compound mean significant differences ($p < 0.05$) among species.

Table S4. Qualitative and quantitative [(mg/kg$_{DW}$ or g/kg$_{DW}$ (marked with *), mean ± SE)] analysis by HPLC-HRMS of the phenolic profile from *in vitro* cultures and micropropagated plants of *Thymus lotocephalus* exposed to different temperatures (30, 25, 20, and 15 °C) for two weeks.

Compound	Treatment							
	In vitro cultures				*Micropropagated plants*			
	30 °C	25 °C	20 °C	15 °C	30 °C	25 °C	20 °C	15 °C
Phenolic acids								
Salvianolic acid A isomer I	<LOQ	<LOQ	<LOQ	<LOQ	<LOQ	<LOQ	<LOQ	<LOQ
*Salvianolic acid A isomer II	0.66 ± 0.00 bcd	0.49 ± 0.03 cd	0.69 ± 0.09 bcd	0.41 ± 0.09 d	0.59 ± 0.13 bcd	0.88 ± 0.07 abc	1.02 ± 0.24 ab	1.19 ± 0.21 a
Salvianolic acid A isomer III	174 ± 6 a	124 ± 22 a	<LOQ	123 ± 15 a	110 ± 24 a	<LOQ	<LOQ	<LOQ
Salvianolic acid A isomer IV	<LOQ	<LOQ	<LOQ	<LOQ	230 ± 12 ab	260 ± 49 ab	329 ± 20 a	194 ± 19 b
Salvianolic acid B /Salvianolic acid L isomer I	179 ± 54 a	182 ± 43 a	207 ± 51 a	180 ± 28 a	155 ± 21 a	194 ± 10 a	176 ± 48 a	152 ± 28 a
Salvianolic acid B /Salvianolic acid L isomer II	151 ± 8 a	126 ±14 a	144 ±21 a	142 ± 29 a	128 ± 19 a	127 ± 18 a	187 ± 43 a	206 ± 37 a
Salvianolic acid B /Salvianolic acid L isomer III	216 ± 5 a	193 ± 23 ab	144 ± 24 ab	115 ± 20 b	<LOQ	<LOQ	<LOQ	<LOQ
Salvianolic acid B / Salvianolic acid L isomer IV	251 ± 1 b	213 ± 23 bc	<LOQ	138 ± 25 c	232 ± 49 bc	383 ± 32 a	462 ± 18 a	225 ± 7 bc
Salvianolic acid C	<LOQ	<LOQ	<LOQ	<LOQ	133 ± 5	<LOQ	<LOQ	<LOQ
Salvianolic acid F isomer I	n.d.	n.d.	n.d.	n.d.	<LOQ	<LOQ	<LOD	<LOQ
Salvianolic acid F isomer II	138 ± 14 a	180 ± 20 a	132 ± 18 a	122 ± 26 a	134 ± 15 a	137 ± 4 a	131 ± 6 a	<LOQ
Salvianolic acid I / Melitric acid A isomer I	<LOD	<LOD	<LOD	<LOD	<LOD	<LOD	<LOD	<LOD
Salvianolic acid I / Melitric acid A isomer II	208 ± 41 cde	161 ± 2 de	138 ± 12 e	<LOQ	386 ± 5 a	227 ± 31 cd	299 ± 1 b	270 ± 15 bc
Salvianolic acid I / Melitric acid A isomer III	406 ± 8 ab	279 ± 52 cd	184 ± 35 d	284 ± 57 cd	431 ± 11 a	313 ± 6 bc	314 ± 16 bc	231 ± 6 cd
****Total salvianolic acids***	2.39 ± 0.09 abc	1.95 ± 0.22 bcd	1.64 ± 0.23 cd	1.52 ± 0.03 d	2.53 ± 0.26 ab	2.52 ± 0.21 ab	2.92 ± 0.36 a	2.47 ± 0.28 ab
Caffeic acid	34 ± 7 d	59 ± 7 c	31 ± 5 d	44 ± 3 cd	52 ± 5 cd	91 ± 9 b	150 ± 1 a	98 ± 8 b
Fertaric acid	n.d.	n.d.	n.d.	n.d.	n.d.	n.d.	n.d.	n.d.
Melitric acid B isomer I	<LOD	<LOD	<LOD	<LOD	<LOD	<LOD	<LOD	<LOD
Melitric acid B isomer II	<LOD	<LOD	<LOD	<LOD	<LOD	<LOD	<LOD	<LOD
*Methyl 6-O-galloyl-β-D-glucopyranoside	5.13 ± 1.28 ab	2.71 ± 0.26 c	6.19 ± 0.74 a	6.10 ± 0.26 a	7.04 ± 0.26 a	2.84 ± 0.08 c	6.35 ± 0.11 a	3.74 ± 0.47 bc

Methylrosmarinic acid isomer I	68 ± 1 a	87 ± 11 a	125 ± 24 a	89 ± 13 a	<LOQ	<LOQ	<LOQ	<LOQ
*Methylrosmarinic acid isomer II	0.91 ± 0.00 ab	0.77 ± 0.10 bc	1.01 ± 0.12 a	1.00 ± 0.08 a	0.89 ± 0.03 ab	0.57 ± 0.05 c	0.73 ± 0.03 bc	0.56 ± 0.01 c
*Rosmarinic acid	25.4 ± 0.73 b	25.6 ± 1.26 b	31.1 ± 1.12 a	28.4 ± 1.44 a	23.4 ± 0.17 b	17.9 ± 0.28 c	19.2 ± 0.46 c	17.3 ± 0.05 c
*Sagerinic acid	1.89 ± 0.03 b	2.00 ± 0.07 b	2.93 ± 0.06 a	1.81 ± 0.34 b	0.60 ± 0.12 c	0.34 ± 0.04 c	0.36 ± 0.09 c	0.23 ± 0.04 c
Salviaflaside	<LOQ	<LOQ	<LOQ	<LOQ	204 ± 3	<LOQ	<LOQ	<LOQ
Total phenolic acids	35.9 ± 1.96 bc	33.2 ± 1.48 cd	43.0 ± 0.42 a	39.0 ± 1.62 b	34.7 ± 0.32 c	24.3 ± 0.66 e	29.7 ± 0.08 d	24.4 ± 0.21 e
Flavonoids								
Dihydromorelloflavone	233 ± 39 ab	266 ± 2 a	230 ± 34 ab	210 ± 13 ab	191 ± 2 b	235 ± 12 ab	194 ± 2 b	243 ± 15 ab
*Epigallocatechin gallate	7.84 ± 2.24 a	9.16 ± 0.06 a	7.68 ± 1.95 a	5.93 ± 5.33 a	5.33 ± 0.12 a	7.65 ± 0.43 a	5.32 ± 0.31 a	5.53 ± 0.26 a
Luteolin	12 ± 0 c	19 ± 1 ab	12 ± 1 c	13 ± 0 c	21 ± 2 ab	20 ± 1 ab	23 ± 1 a	19 ± 1 b
Luteolin-7-O-glucuronide	132 ± 25 ab	175 ± 3 a	153 ± 4 ab	93 ± 6 b	102 ± 8 ab	158 ± 17 ab	153 ± 48 ab	164 ± 17 ab
*Theaflavic acid	1.22 ± 0.08 a	1.22 ± 0.11 a	1.35 ± 0.10 a	1.26 ± 0.00 a	0.86 ± 0.00 b	0.82 ± 0.03 b	0.79 ± 0.00 b	0.76 ± 0.01 b
Total flavonoids	9.44 ± 2.15 ab	10.8 ± 0.17 a	9.42 ± 2.02 ab	7.50 ± 0.53 ab	6.51 ± 0.12 b	8.89 ± 0.37 ab	6.48 ± 0.26 b	6.71 ± 0.22 b
Coumarin derivative								
Herniarin	<LOD	<LOQ	<LOQ	<LOD	n.d.	n.d.	<LOD	<LOD
Hydroxybenzaldehyde								
Protocatechuic aldehyde	76 ± 13 b	81 ± 1 b	58 ± 10 b	87 ± 3 b	84 ± 19 b	99 ± 20 b	152 ± 20 a	91 ± 1 b
Total phenolic compounds	45.4 ± 4.12 b	44.1 ± 1.31 b	52.5 ± 2.44 a	46.6 ± 2.15 ab	41.3 ± 0.42 bc	33.2 ± 1.01 d	36.3 ± 0.37 cd	31.2 ± 0.43 d

Notes: n.d. – not detected; LOD – limit of detection; LOQ – limit of quantification. The results were analyzed using a one-way analysis of variance (ANOVA) followed by Duncan's New Multiple Range Test. Distinct letters (a to e) in each row and for each phenolic compound mean significant differences ($p < 0.05$) among species.
